# Severe experimental folate deficiency in a human subject – a longitudinal study of biochemical and haematological responses as megaloblastic anaemia develops

**DOI:** 10.1186/2193-1801-3-442

**Published:** 2014-09-23

**Authors:** Paul Henry Golding

**Affiliations:** Unit 5, 18 Webster Road, Nambour, QLD 4560 Australia

**Keywords:** Experimental folate deficiency, Megaloblastic anaemia, Red-cell folate, Liver folate, Folate kinetics and metabolism, Folate model, Folate immunoassays, Immunoassay errors, Self-experimentation

## Abstract

**Background:**

The currently accepted theory, that the human liver store of folate is limited to about four months, is based on the findings of Victor Herbert and others of the era before folate fortification of food. A recent model, developed by Lin et al., predicts far greater liver folate storage capacity than reported by Herbert. The conflict between Herbert’s and Lin’s models needs to be resolved experimentally, however current research is restricted because ethical considerations prevent such risky experimentation on patients or healthy human volunteers. The objective was to provide a detailed record of the biochemical and haematological responses to the development of severe experimental folate deficiency in an initially replete human subject.

**Methods:**

This 58 year old male severely depleted himself of folate, using a folate-deficient diet, until overt megaloblastic anaemia developed. The biochemical and haematological responses were monitored by routine blood tests. Daily intake of dietary supplements prevented deficiencies of other relevant nutrients.

**Results:**

The rate of change of all analytes was significantly slower, and the delay before any change for several analytes was significantly longer, than reported for previous experiments. The time before reporting of abnormal biochemical and haematological results was therefore very significantly longer than reported by Herbert, but was consistent with the recent model of Lin et al. Serum folate and red-cell folate became abnormally low after 219 and 413 days respectively. Macrocytic anaemia was produced after 469 days, and megaloblastic anaemia was confirmed by bone marrow biopsy on day 575. Folate starvation ceased on day 586, and recovery was complete on day 772.

**Conclusions:**

The currently accepted four month time scale for development of megaloblastic anaemia from folate deficiency, based on the early work of Herbert and others, is not consistent with the results from this study. The > 300 day liver folate storage time, predicted by the model of Lin et al., is supported by this experiment. Self-experimentation has produced a detailed record of the biochemical and haematological responses to severe experimental folate deficiency, whereas using patients or healthy volunteers as subjects would be unethical.

**Electronic supplementary material:**

The online version of this article (doi:10.1186/2193-1801-3-442) contains supplementary material, which is available to authorized users.

## Background

Victor Herbert used self-experimentation to prove that dietary folate deficiency could cause megaloblastic anaemia (Herbert 1962, 1963; Lichtman et al. 2000). From that study, he developed his hypothesis for the sequential stages in the development of folate deficiency (Herbert 1964, 1987a), and recommended dietary folate intakes (Herbert 1987b). Potassium and iron deficiencies, caused by the special diet, confounded the quantitative haematological results (Herbert 1962; Lindenbaum et al. 1995; Stabler 2009). Other early studies of dietary folate deficiency culminating in anaemia were performed on healthy volunteers, alcoholics and cancer patients (Eichner et al. 1971a; Eichner et al. 1971b; Gailani et al. 1970). All of these early experiments were restricted, in the range of haematological and biochemical parameters measured, by available technology. For example, automated cell counters were not yet available. More recent studies of dietary folate deficiency have been terminated before the onset of megaloblastic anaemia (Lindenbaum et al. 1995; Sauberlich et al. 1987; Milne et al. 1983; Kauwell et al. 2000; Jacob et al. 1994; Jacob et al. 1998).

The study of folate kinetics and one-carbon metabolism is important for understanding the role of folate, and in assessing folate status and preventing folate deficiency (Gregory et al. 2009; Lin et al. 2004; Pietrzik et al. 2007; von der Porten et al. 1992; Gregory and Quinlivan 2002; Gregory et al. 1998; Stites et al. 1997). Biochemical and haematological response data, from studies of experimental dietary folate deficiency in humans, provides valuable inputs into models of folate metabolism (Herbert 1987a; Lindenbaum et al. 1995; Pietrzik et al. 2007). Gregory et al. ( 2009) noted the very marked differences between models.

The findings of Herbert, and others of the pre-folate-fortification era, suggest that the liver store of folate is limited to about four months. Herbert ( 1987b) states: “*Red cell folate reflects liver folate fairly closely ....by a coincidence of nature, the red cell life-span is 4 mo and the liver-folate stores will last for 4 mo*.”

Herbert’s hypothesis, supported by his own observations and those of other researchers of the time, was widely accepted as the model for the development of folate deficiency. According to Hoffbrand and Weir ( 2001): “*Victor Herbert performed the famous experiment in which he ate a folate-deficient diet for 4 months and monitored the sequence of events, haematologically and by assay for folate in blood. He showed that it took about 4 months for megaloblastic anaemia to develop.*” Current advice continues to be based on the findings of the researchers of the pre-folate-fortification era. For example, Hoffbrand and Provan ( 2007) state that: “*Body stores are sufficient for only about four months.*”

A recent model, developed by Lin et al. ( 2004), predicts far greater liver folate storage capacity than reported by Herbert. Due to enterohepatic recycling, the actual size of liver folate storage will significantly affect the time taken for the onset of folate deficiency following commencement of folate deprivation. Lin found that his model predicted body stores of folate to be nearly five times greater than previously published and that these stores would last for hundreds of days when on a folate-deficient diet.

There have been very few longitudinal studies where megaloblastic anaemia has been produced, and none since the commencement of folate fortification of food, because of ethical concerns regarding risk in human experimentation when using patients or healthy volunteers as subjects (Lichtman et al. 2000; Lindenbaum et al. 1995). Self-experimentation has previously been used successfully in medical research in general (Altman 1998; Weisse 2012), and nutrition research in particular (Widdowson 1993), to overcome any ethical concerns.

In 2009, while monitoring for a possible secondary folate deficiency during a self-experiment to investigate tests for vitamin B_12_ deficiency, this author discovered very significant differences between results from three red-cell folate immunoassay systems. Commencing in May 2011, this author used himself as the subject of an experiment to investigate these discrepancies. During that investigation, described in detail in a separate report (Golding 2014), the biochemical and haematological responses were recorded.

### Objective

The objective of this study was to provide a detailed record of the biochemical and haematological responses to the development of severe experimental folate deficiency, in an initially folate-replete human subject, by means of self-experimentation.

## Methods

### Ethics statement

As a member of COPE (Committee on Publication Ethics), SpringerPlus requires that experiments on human subjects adhere to the ethical standards of the Declaration of Helsinki. In particular, there must be informed consent of subjects, and the experiment must be approved and overseen by a research ethics committee or institutional review board. Because this author did not obtain informed consent, and the study did not receive ethics committee approval, it is necessary for the author to explain the reasons why publication of this report is ethical.

Firstly, the author was both the experimenter and the single subject, so the requirement for informed consent does not apply. There was no institutional involvement, so there was no possibility of coercion. The subject was assessed by a psychiatrist before the experiment commenced, and found to be competent to evaluate the risks and benefits, and to accept full responsibility for the conduct of the experiment.

Secondly, the Declaration of Helsinki is silent on self-experimentation, because it is concerned with the conduct of research on patients or healthy volunteers by others. The requirement for ethics committee approval therefore does not apply where the single subject is also the sole experimenter. Also, because there was no institution involved in the study, with the experiment conducted by an independent researcher, no ethics committee existed.

Thirdly, the experiment was not performed recklessly or carelessly; the subject’s condition was monitored weekly by the psychiatrist. This doctor is a Fellow of the Royal Australian and New Zealand College of Psychiatrists, and had no conflict of interests. Being a qualified medical practitioner receiving all weekly pathology reports, he was able to continually assess the condition of the subject. For safety, it was agreed at the outset that he would take control if, but only if, there was an immediate life-threatening condition. The subject instigated, designed and performed the experiment, and this doctor’s only role was monitoring for safety.

Lastly, the motivation for performing the experiment was ethical, and involved no conflict of interests. The author wanted to investigate the gross differences between the red-cell folate immunoassays, as found during his experiment of 2009, because he was aware of the potential consequences of errors in measurement of folate concentrations, especially in pregnant women and others at high risk of deficiency. The author was motivated only by the desire to gain and share knowledge, to advance medical science, for the benefit of patients.

### Experiment design

A human subject, initially replete in folate, consumed a folate-deficient diet to severely deplete the body of folate, culminating in megaloblastic anaemia. Precautions were taken to avoid the confounding effects of other nutrient deficiencies. The biochemical and haematological responses were monitored by weekly or twice-weekly blood tests during both the depletion and recovery stages. Three commercial clinical pathology laboratories, all accredited by the Australian National Association of Testing Authorities (NATA), were used to increase confidence in the results. One research laboratory, specializing in microbiological folate assays, was used to confirm severe folate depletion of the subject. Megaloblastic anaemia was confirmed by blood cell counts, blood films, and bone marrow examination.

### The subject

The subject was this author, a 58 year old male non-drinker, with no history of folate deficiency or anaemia. He had for many years consumed a vegetarian diet replete in folate, and had never taken folate supplements. There are no reported interferences, with any of the analytes monitored, from any medication taken by the subject. Because of a history of vitamin B_12_ deficiency, of uncertain cause, the subject had been taking 1000 μg oral methylcobalamin daily for two years immediately prior to this study. Extensive testing showed consistently normal results for serum vitamin B_12_ and the two metabolites, homocysteine and methylmalonic acid, at this level of intake.

### The folate-deficient diet

**Figure 1:**
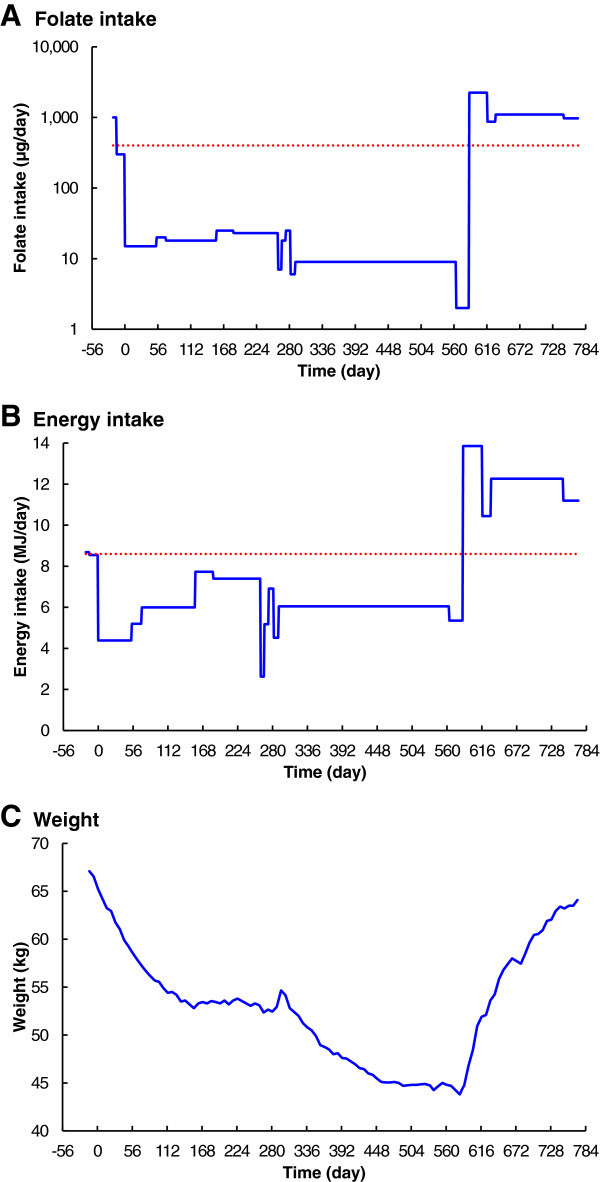
**Changes in folate and energy intake, and body weight over time. A**. Folate intake. **B**. Energy intake. **C**. Weight. Severe dietary folate deficiency commenced on Day 0 (23 May 2011). The folate-replete diet, with folate supplementation, resumed on day 586 (29 December 2012), and recovery was complete on day 772 (3 July 2013). The dotted line in panel **A** is the recommended daily intake for folate (Institute of Medicine, National Academy of Sciences 1998). The dotted line in panel **B** is the initial estimated daily energy requirement for the subject (Australian Government National Health and Medical Research Council 2013a).

The folate-deficient diet was a modified vegetarian version of that used by Herbert ( 1962, 1963). Several variations were made during the course of the experiment to improve the balance between folate reduction and energy maintenance (Figure 1A and B). This was necessary to minimize the significant weight loss (Figure 1C) caused by the semi-starvation diet. The average daily dietary folate intake, during the depletion stage, varied from 25 μg to 2 μg, or 6% to 0.5% of the recommended daily intake (RDI) of 400 μg (Institute of Medicine, National Academy of Sciences 1998). The folate concentration for each food item was taken from the data provided by Food Standards Australia and New Zealand ( 2011). For the last ten months of folate starvation, the diet consisted primarily of white rice, boiled and washed three times, flavoured with either salt or coconut and sugar; additional energy was supplied by Gatorade and barley sugar. Compliance with the diet was 100%.

### Nutritional precautions

#### Methionine

Because methionine deficiency could limit the production of homocysteine, the subject consumed methionine supplements (Musashi L–Methionine). This was taken at a rate of 370 mg per day, from day 77 to day 279, during a methionine-deficient variant of the low-folate diet. The quantity of methionine supplement was calculated to be half the RDI, of 740 mg/d based on the subject’s body weight, according to the UN FAO ( 1991). This amount was chosen to avoid any “methionine loading” effect on the homocysteine concentration (Connor et al. 1978). In a healthy subject, with no nutrient deficiency, homocysteine concentration is not dependent on methionine intake (Ward et al. 2000; Shimakawa et al. 1997), but this was not assumed to apply in the case of a folate deficiency.

#### Iron

The subject daily consumed 325 mg ferrous sulphate, equivalent to 105 mg elemental iron, with 562 mg sodium ascorbate (Abbott Australia Ferro-Grad C), to prevent the iron deficiency reported by Herbert ( 1962). The sodium ascorbate (vitamin C) is used to assist in the absorption of the iron. According to the Australian Government National Health and Medical Research Council ( 2013b), the iron RDI for men is 8 mg/day. The subject has no known defect in iron absorption, so this iron supplement should have ensured adequate iron status. To check for any deficiency, iron studies were performed twice during the folate-starvation stage of the experiment.

#### Potassium

The subject daily consumed 1800 mg slow-release potassium chloride (Novartis Slow-K) to prevent the potassium deficiency reported by Herbert ( 1962). Serum potassium concentrations were routinely monitored during the experiment.

#### Vitamins

To avoid vitamin B_12_ deficiency, the subject continued to take 1000 μg methylcobalamin daily and, as a precaution against deficiencies of other B-group vitamins, he also consumed 62 mg of vitamin B_3_, and 12 mg of each of vitamins B_1_, B_2_, and B_6_ daily.

### Blood sampling

#### Blood sample collection

Precautions were taken to ensure that consistent and valid blood samples were received by the laboratories. Each of the three laboratories that performed the routine biochemistry and haematology tests collected their own samples, using professional phlebotomists at government-approved collection centres. In the morning of each sampling day, the blood samples were all collected within the narrowest possible time window; usually not more than two hours from first to last. The subject always fasted overnight, and was well hydrated, ensuring maximum possible consistency between samples. The phlebotomy technique use by each collector was chosen to provide the highest quality samples; tourniquet application was carefully controlled, and discard tubes were used where required. All samples were promptly transported from the collection centre to the laboratory, cooled on ice, to avoid deterioration.

#### Blood sampling frequency

The frequency of routine blood sampling was adapted according to the rate of change of the biochemical and haematological responses. Blood samples were initially collected weekly; this was increased to twice weekly for the last three months of the folate depletion stage and the first month of the recovery stage.

### Folate immunoassays

#### Immunoassay red-cell folate

All three commercial clinical pathology laboratories were used to assay red-cell folate, using automated immunoassay systems. Laboratory A used the Siemens Advia Centaur; Laboratory B used the Roche Elecsys 2010; Laboratory C used the Beckman UniCel DxI 800.

#### Immunoassay serum folate

Laboratories A and B reported serum folate using the same systems used for red-cell folate; Laboratory C did not report serum folate.

### Folate microbiological assays

Microbiological serum and red-cell folate assays, of a single blood sample collected on day 574, were used to confirm severe folate depletion of the subject. The assays were performed by the Food Science and Human Nutrition Department, University of Florida. The procedure, based on that described by O'Broin and Kelleher ( 1992), was developed by the Centers for Disease Control and Prevention (Zhang et al. 2008).

Special precautions were taken, during collection and transport of the blood samples, to ensure that valid samples were received by the laboratory. Serum samples were collected in serum separator tubes, and whole blood samples were collected in EDTA tubes. The serum tubes were spun down at the collection centre, and all samples were immediately transported, cooled on ice, to the local laboratory. Aliquots of serum and whole blood were then transferred to cryovials and frozen to −80°C. The samples were sent, packed in dry ice, to the laboratory in Florida by specialized international courier. All samples were received frozen, and in excellent condition, then stored at −80°C until assayed.

### Other biochemistry assays

#### Homocysteine

Serum total homocysteine was routinely assayed by Laboratory A only, using the Siemens Advia Centaur immunoassay system.

#### Bilirubin and lactate dehydrogenase

Liver functions, including total bilirubin and lactate dehydrogenase, and electrolytes were assayed by Laboratory B using the Roche Cobas Integra 400 Plus.

#### Iron

Iron studies were performed by Laboratory C, during the folate-starvation period, on days 420 and 574. Serum iron, transferrin IBC, transferrin saturation and serum ferritin were all reported.

#### Vitamin B_12_

Vitamin B_12_ was assayed by Laboratory B, once at the commencement of the experiment and then four times during the folate-starvation period, using the Roche Elecsys 2010.

### Haematology

#### Cell counts

Three clinical pathology laboratories, each equipped with commercially available automated cell counters, were used to monitor the haematological responses to folate depletion. Laboratories A and C used the Sysmex XE-2100 Automated Haematology System; Laboratory B used the Sysmex XS-1000i haematology analyser.

#### Bone marrow examinations

Two bone marrow biopsies were performed by an experienced physician, and samples were forwarded to Laboratory C for examination; the first on day 402; the second on day 575. Whole blood, bone marrow aspirate and trephine samples were collected. Marrow aspirate was examined for cellularity, myelogram was performed, and surface markers analysed. Marrow trephine was examined for normality of bone trabeculae, cellularity and infiltration.

### Recovery diet

The diet used for the recovery stage initially contained more than 2000 μg folate daily, comprising natural folates and folic acid fortification (Figure 1A). An additional 400 μg folic acid supplement was taken daily for the duration of the recovery stage.

## Results

### Data availability

**Figure 2:**
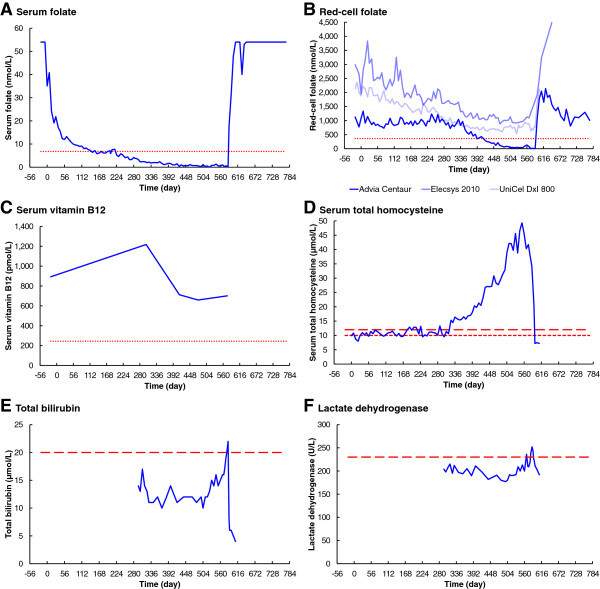
**Changes in biochemistry over time. A**. Serum folate. **B**. Red-cell folate. **C**. Serum vitamin B_12_. **D**. Serum total homocysteine. **E**. Total bilirubin. **F**. Lactate dehydrogenase. The dotted lines in panels **A** to **C** are the minimum normals according to Bates and Lewis (2012). The short-dashed line in panel **D** is the maximum limit for patients with high coronary risk factors (Stanger et al. 2004), and the long-dashed line in panel **D** is the maximum limit in case of normal coronary risk factors (Brouwer et al. 1998). The dashed lines in panels **E** and **F** are maximum normals quoted by the testing laboratory.

**Figure 3:**
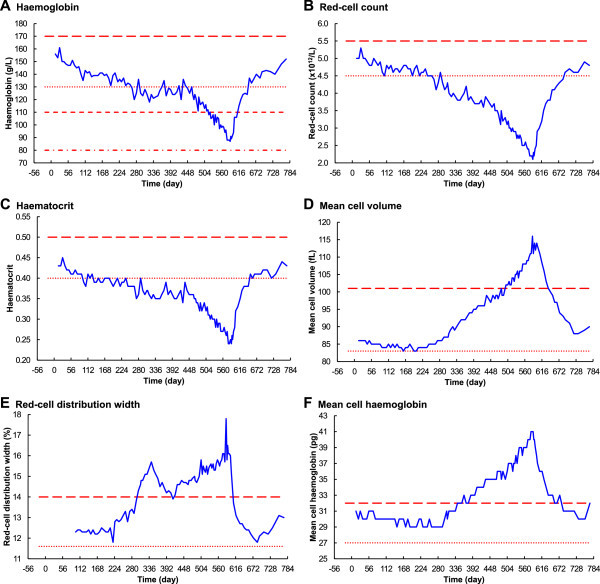
**Changes in haematology over time – part 1. A**. Haemoglobin. **B**. Red-cell count. **C**. Haematocrit. **D**. Mean cell volume. **E**. Red-cell distribution width. **F**. Mean cell haemoglobin. The dotted lines are the minimum normals, and the long-dashed lines are the maximum normals, according to Bates Bates and Lewis (2012). The dotted line in panel **A** is also the WHO haemoglobin level for mild anaemia; the short-dashed line is the WHO level for moderate anaemia and the dot-dashed line is the WHO level for severe anaemia (World Health Organization 2011).

**Figure 4:**
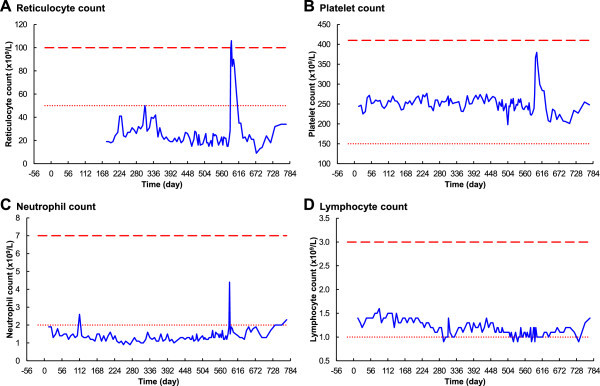
**Changes in haematology over time – part 2. A**. Reticulocyte count. **B**. Platelet count. **C**. Neutrophil count. **D**. Lymphocyte count. The dotted lines are the minimum normals, and the long-dashed lines are the maximum normals, according to Bates and Lewis (2012).

**Figure 5:**
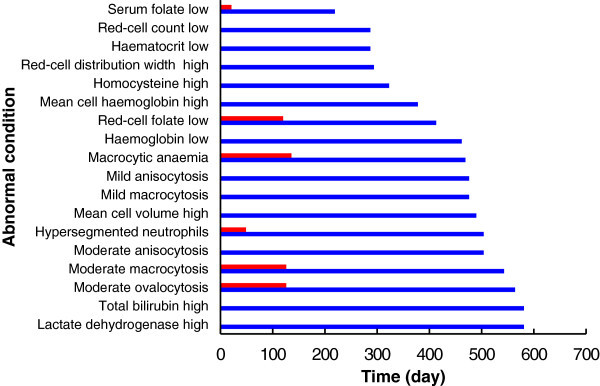
**Sequence of abnormal events.** For quantitative data, the blue bars show the time taken for consistently abnormal results to be reported. For examination of blood-film slides, the blue bars show the time before the first report of abnormality. The folate results used here are from Laboratory A, using the Siemens Advia Centaur. The red bars are the results reported by Herbert (1962).

**Figure 6:**
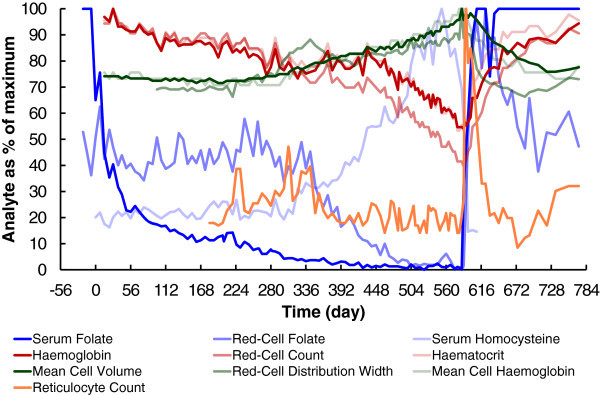
**Comparison of changes in analytes over time.** All analytes are shown as a percentage of their maximum value reported for the duration of the experiment. The folate results used here are from Laboratory A, using the Siemens Advia Centaur.

The data sets supporting all results are included in a Microsoft Excel spreadsheet file, Additional file 1, containing charts and tables. Details of calculations for the red-cell lifespan are available, as a Microsoft Excel spreadsheet, in Additional file 2. High-resolution images for Figures 1 to 6 are included in a PDF file, Additional file 3, and a Microsoft PowerPoint file, Additional file 4.

### Laboratories

The immunoassay red-cell folate results from all three laboratories have been presented here, because they differ very significantly. The immunoassay serum folate results are those from Laboratory A only. Laboratory B produced similar results, but discontinued reporting serum folate before the folate depletion stage of the experiment was concluded; Laboratory C did not report any serum folate results.

The haematology results presented here are from the primary clinical pathology laboratory, Laboratory A, only. To improve clarity and, as there were not generally significant differences between them, the haematology results from Laboratories B and C have been omitted from the charts.

### Folate immunoassays

#### Immunoassay serum folate

Serum folate (Figure 2A) was initially above the analyser limit of 54 nmol/L, a presumed consequence of an initial folate-replete diet. Serum folate responded without delay, after commencement of the folate-deficient diet on Day 0, rapidly falling to a stable level near the normal lower limit of 6.8 nmol/L in 147 days. There was no consistent fall below normal until after day 219, when it fell slowly but consistently to reach zero on day 525. Serum folate increased rapidly immediately after restoration of the normal folate-replete diet.

#### Immunoassay red-cell folate

The three clinical pathology laboratories reported very significantly different results for red-cell folate (Figure 2B). Results from Laboratory A initially vary randomly around 1000 nmol/L, with no significant response until day 343. There is then a decelerating fall, to below the normal lower limit of 360 nmol/L on day 420, reaching zero folate on day 574. Red-cell folate results from Laboratory B show an initial concentration of 2988 nmol/L, falling slowly but without significant delay and with initial severe instability, reaching a minimum of 879 nmol/L on day 469. Laboratory C shows an initial red-cell folate concentration of 2142 nmol/L, falling slowly but without significant delay, with slight instability, reaching a minimum of 535 nmol/L on day 525. All three laboratories reported a rapid increase in red-cell folate levels immediately following resumption of the folate-replete diet.

### Folate microbiological assay

The Food Science and Human Nutrition Department, University of Florida, provided a summary report of their results for red-cell folate and serum folate (unpublished; copy available from this author) for samples collected on day 574. The mean serum folate was 0.6 ± 0.09 nmol/L (n = 8), with an inter-assay CV of 5.4% and an intra-assay CV of 7.9%. The mean red-cell folate was 99 nmol/L, with an inter-assay CV of 12.5% and an intra-assay CV of 7.2%. The report noted: “*We have never analyzed samples with folate concentrations as low as these samples.*”

### Other biochemistry assays

#### Homocysteine

Serum total homocysteine was initially 9.9 μmol/L, varying around the recommended upper limit of 10.0 μmol/L, but with no significant change until day 323 (Figure 2D). The homocysteine concentration then increased exponentially, with some short-term variations, to reach a maximum of 49.3 μmol/L on day 553. Following the resumption of the folate-replete diet, the serum homocysteine concentration fell rapidly to 7.2 μmol/L.

#### Bilirubin and lactate dehydrogenase

Total bilirubin and lactate dehydrogenase (LD) were not monitored until day 295 (Figure 2E and F). Total bilirubin showed some random short-term variations, below the normal upper limit of 20 μmol/L until day 518. The bilirubin concentration then increased exponentially, to above the normal range, reaching a maximum of 22 μmol/L on day 585. LD showed a slow and insignificant fall, from 204 to 177 U/L, until day 498. The LD concentration then increased exponentially, to above the normal upper limit of 230 U/L, reaching a maximum of 252 U/L on day 585. Total Bilirubin and LD rapidly fell, back into the normal range, after resumption of the folate-replete diet.

#### Iron

Results of the two iron studies performed during the folate-starvation period were all within the normal range for the laboratory. Although iron stores were reported as mildly depleted in the first bone marrow sample collected on day 402, serum iron, transferrin IBC, transferrin saturation and serum ferritin were all reported as normal 18 days later. All iron studies results were again reported as normal on day 574, and marrow iron stores were normal on day 575, 11 days before the end of the folate-starvation period.

#### Vitamin B_12_

Serum vitamin B_12_ remained above the normal lower limit of 244 pmol/L throughout the experiment (Figure 2C). There was some variation; 893 pmol/L initially, peaking at 1218 pmol/L on day 301, then falling to a minimum of 659 pmol/L on day 476.

### Haematology

#### Haemoglobin, red-cell count and haematocrit

Haemoglobin, red-cell count and haematocrit were all within their normal ranges at the commencement of the experiment (Figure 3A to C). All three analytes initially responded similarly to folate depletion, without significant delay, falling slowly until about day 323. Haemoglobin and haematocrit then remained without significant change until about day 469. Red-cell count behaved differently, continuing to fall at a reduced rate, until about day 469. Haemoglobin, red-cell count and haematocrit all then fell linearly, to very significantly below the normal range, reaching minimum levels near day 588, two days after resumption of the folate-replete diet. They all then increased without further delay, returning to their initial levels on day 772.

#### Mean cell volume, red-cell distribution width and mean cell haemoglobin

Mean cell volume (MCV), red-cell distribution width (RDW) and mean cell haemoglobin (MCH) were initially all within their normal ranges (Figure 3D to F). MCV fell slightly after commencement the folate deficient diet, remained steady around 84 fL until day 238, then increased exponentially to a maximum of 116 fL on day 585. Similarly, MCH fell slightly, remained steady around 29 pg until day 301, and then increased exponentially to a maximum of 41 pg on day 585. RDW behaved differently, having two distinct peaks at days 343 and 585. MCV, RDW and MCH all rapidly fell without delay, into their normal ranges, after resumption of the folate-replete diet.

#### Reticulocyte count

Reticulocyte count was initially low at 19 × 10^9^/L, reached a peak of 50 × 10^9^/L on day 308, then fell and varied between 15 × 10^9^/L and 28 × 10^9^/L until day 588 (Figure 4A). Reticulocyte count rapidly increased to reach 99 × 10^9^/L on day 590, after resumption of the folate-replete diet on day 586; there was then a short-term peak at 106 × 10^9^/L, just above the maximum limit of the normal range.

#### Platelets, neutrophils and lymphocytes

The platelet count did not respond to the onset of the folate-deficient diet, remaining in the normal range until day 585 (Figure 4B). The neutrophil count was initially borderline low, at 1.9 × 10^9^/L, decreased slowly to a minimum of 0.9 × 10^9^/L on day 252, then increased slowly to reach 1.4 × 10^9^/L on day 585 (Figure 4C). After resumption of the normal folate-replete diet there was a very rapid response of both platelets and neutrophils; platelets peaked at 390 × 10^9^/L on day 599, and neutrophils peaked at 4.4 × 10^9^/L on day 590. The Lymphocyte count slowly decreased over the depletion stage, then remained at borderline-low levels. After a significant delay, following resumption of the folate-replete diet, lymphocyte numbers rapidly returned to normal by day 772 (Figure 4D).

#### Bone marrow examinations

The results of the first bone marrow examination were all normal except for mildly decreased iron stores, whereas those for the second clearly demonstrated megaloblastic anaemia. Cellularity of the aspirate was moderately increased, erythropoiesis showed moderately dyserythropoietic features, including megaloblastic changes and poor hemoglobinization. Some hypersegmented neutrophils were present but lymphocytes were normal. Plasma cells were normal and megakaryocytes were within normal limits; there was an occasional very large form with bizarre nuclear morphology. Iron stores were normal; an occasional ring sideroblast was present. Bone marrow surface markers were all normal. Trephine biopsy marrow was hypocellular in one half and normal in the other; trabeculae appeared normal. There was no evidence of malignant infiltration.

### The subject

The subject’s weight fell very severely, from 67 kg to 44 kg, during the depletion stage of the experiment (Figure 1C), and hunger was often severe. He became extremely tired and weak, with periods of irritability, confusion, and sleeplessness. Unlike Herbert ( 1962), this subject did not experience euphoria on resumption of the folate-replete diet.

## Discussion

The primary finding of this part of the experiment was the very long time required, from commencement of folate-starvation, to develop megaloblastic anaemia. The rate of change of all analytes was significantly slower, and the delay before any change for several analytes was significantly longer, than reported by previously by Herbert and others (Herbert 1962; Eichner et al. 1971a; Eichner et al. 1971b; Gailani et al. 1970) (Figures 5 and 6). The time before reporting of abnormal biochemical and haematological results was therefore very significantly longer than expected. Both serum and red-cell folate became consistently abnormally low after 219 and 413 days respectively for this subject, whereas Herbert achieved these low levels in only 21 and 120 days. Macrocytic anaemia was first reported after 469 days for this subject, compared to 126 days for Herbert ( 1962).

According to Herbert ( 1964), the time taken to develop a deficiency of red-cell folate, following cessation of folate intake, is dependent on the lifespan of red blood cells because folate cannot enter or leave mature erythrocytes. Also according to Herbert ( 1964, 1987a), the lifespan of red blood cells is 120 days, after which all of the original folate-replete erythrocytes will have been removed from circulation.

There have been several attempts to measure the lifespan of red blood cells in humans (Franco 2009; Furne et al. 2003; Mitlyng et al. 2006). Recent measurements, by Furne et al. ( 2003) using breath carbon monoxide, gave 122 ± 23 days in 40 healthy volunteers. The lifespan is affected by chronic disease, often significantly reduced (Mitlyng et al. 2006), but this author has found no reported cases of greatly extended erythrocyte lifespan.

Laboratories B and C reported red-cell folate falling from day 0, suggesting a red-cell lifespan of greater than the 585 days of folate starvation. Based on the results of Furne et al. ( 2003), the probability of a subject having a red-cell lifespan longer than 585 days is less than 1 in 10^30^. Details of this calculation are available, as a Microsoft Excel spreadsheet, in Additional file 2. It is therefore reasonable to exclude an abnormally long red-cell lifespan, for this subject, as an explanation for the unexpectedly long time taken to deplete the folate store.

According to Laboratory A, red-cell folate commenced falling below the baseline concentration after day 343 and reached 0 nmol/L on day 574. Homocysteine and MCV also commenced increasing above their baseline concentrations after about day 300. These results suggest an initial delay of about 300 days, before there is any reduction of folate available to the red-cells, followed by about 230 to 270 days during which the folate was depleted during erythrocyte turnover. Again, based on the results of Furne et al. ( 2003), the probability of a subject having a red-cell lifespan longer than 230 days is about 1 in 10^6^. Although this subject could have had such an abnormally long red-cell lifespan, it is not the most likely explanation for the unexpectedly slow rates of change.

Lin’s model (Lin et al. 2004), showing far greater liver folate storage capacity than reported by Herbert, offers a reasonable explanation; the liver folate store can last far longer than 120 days. By means of enterohepatic recycling, the red-cell folate level is maintained until the liver stores are exhausted. This recycling of folate, with a large liver store, would explain the initial 300 day delay before many analytes changed significantly from their baseline concentrations, as well as the greater than 230 days then required to totally deplete the red-cell folate.

Herbert ( 1987a, 1987b) stated that the liver coincidentally stores a four month supply of folate, thus explaining his observation that both red blood cells and the liver are simultaneously depleted of folate after this time. Because his experiment was performed in the time before folate fortification of food, it is likely that Herbert, and other subjects of his era, had comparatively small initial liver folate stores. This subject had consumed a diet replete in folate-fortified food, for many years before commencement of the experiment, so his liver folate store is likely to have contained a much larger amount of folate than Herbert’s.

The practical experimental data presented here, together with the model of Lin et al. ( 2004), proves that Herbert’s assumption that liver storage is limited to 120 days was incorrect. In a truly folate-replete individual, of the post-fortification era, liver stores can last far longer than four months. This experiment demonstrates that the time taken for folate deficiency to occur, from the commencement of folate starvation, can be much longer than the 120-day life span of the red cells.

### Potential confounding factors - pure folate deficiency

Stabler ( 2009) refers to the difficulty of obtaining pure folate deficiency: “*Other major causes such as malabsorption and food deprivation also lead to multiple nutrient deficiencies; thus, it has been difficult to study “pure” cases of folate deficiency.*”

It is not possible, in the real world of practical experimental medicine, to achieve 100% “pure” folate deficiency. To define such a condition, let alone actually achieve it, poses many insoluble problems, because it would require: a perfect ideal model average human subject, with no history of any medical condition; a fully controlled perfect environment; a perfect diet with total control over all nutrients.

Such a condition of “pure” folate deficiency has never been achieved, and never will be, except in theoretical models. What is important is to consider the major potential confounding factors, where possible to avoid them, and take into account any that cannot be avoided.

This author does not claim to have achieved a 100% pure model of folate deficiency. The experiment has produced the first detailed longitudinal study of the development of folate deficiency in a human subject since the folate fortification of food. The introduction of modern automated cell counters has also provided far more data than was available to the earlier researchers of Victor Herbert’s era.

The potential effect of confounding factors also needs to be considered in the context of the purpose and findings of the experiment. The primary finding in this study, that the time taken to achieve megaloblastic anaemia for this subject was far longer than for previous experiments, was not affected by any known confounding factor.

#### Methionine deficiency

The methionine supplements ensured that methionine deficiency did not confound the results.

The absence of any effect on the homocysteine concentration, at the commencement or ceasing of the methionine supplement, together with the expected rapid increase in homocysteine concentration on depletion of folate stores, confirms that the potential confounding effect of methionine deficiency was avoided.

#### Protein-energy deficiency

Stabler ( 2009) refers to the potential confounding effect of protein-energy deficiency: “*Protein-calorie malnutrition may cause serious fat atrophy of the bone marrow and hypoplasia, which could mask megaloblastic changes observed in folate deficiency.*”

Liver proteins and creatinine were tested many times during the experiment. Although proteins and creatinine were reported as ranging from borderline to low, no fat atrophy or hypoplasia were reported in either of the bone marrow examinations of days 402 and 574. The first marrow examination reported no abnormalities; this confirms that the low-protein diet did not cause any significant confounding effects. The second marrow examination reported *“obvious erythroid hyperplasia”*, clearly demonstrating the effect of folate deficiency.

#### Iron deficiency

According to Stabler ( 2009), iron deficiency can confound the effect of folate deficiency: “*Iron deficiency, characterized by a reduction in MCV, caused by bleeding or consumption of an iron-deficient diet that is common in those with alcoholism, will mask the macrocytosis of folate deficiency.*”

As confirmed by the results of the iron studies, and the second bone-marrow examination, the iron supplements ensured that there was no iron deficiency. Furthermore, there was no evidence of any confounding effect of iron deficiency. The subject's mean cell volume was never below the reference intervals for the three testing laboratories used, and abnormalities due to iron deficiency were never reported on their weekly blood films. In addition, the MCV increased as expected when folate deficiency progressed.

#### Vitamin B_12_ supplements

Although it is well known that large doses of folate can reduce the haematological effects of vitamin B_12_ deficiency, this author was unable to find any published studies where vitamin B_12_ supplements have masked all of the effects of a primary folate deficiency.

Furthermore, the results show that there was no actual confounding effect of the vitamin B_12_ supplementation. With the vitamin B_12_ intake constant throughout the experiment, the effects of the folate deficiency can be clearly observed in the charts for biochemistry and haematology. For example, the homocysteine ranged from normal to a very high peak; any significant effect of the vitamin B_12_ supplement would be expected to suppress this.

### Limitations of the experiment

There was only one subject, however such a study can disprove a currently accepted theory. As stated by Stephen Hawking ( 1988): “*Any physical theory is always provisional, in the sense that it is only a hypothesis: you can never prove it. No matter how many times the results of experiments agree with some theory, you can never be sure that the next time the result will not contradict the theory. On the other hand, you can disprove a theory by finding even a single observation that disagrees with the predictions of the theory.*”

In the past few decades there has been an increasing trend towards large trials, involving many subjects, with numerous authors funded by large institutions. Despite this move away from small scale research, independent single-subject experiments can continue to contribute to medical science. According to Allen B. Weisse ( 2012), “*many self-experiments have proved invaluable to the medical community and to the patients we are seeking to help.*”

A highly relevant example of a worthwhile single-subject experiment is the study by the late Victor Herbert (Herbert 1962), whose work was so significant that the report was re-published as a landmark haematology paper of the 20th century (Lichtman et al. 2000). There are numerous other examples of significant contributions made to medical science by single-subject experiments (Altman 1998; Weisse 2012; Widdowson 1993).

For ethical reasons, a study such as this one can only be performed by means of self-experimentation on a single subject. It is therefore not possible to measure the distribution of times, for development of megaloblastic anaemia from folate deficiency, in a large group of subjects. What is important is that the primary result of this experiment, that a single subject had a nineteen month supply of folate, disproves the currently accepted theory that the liver store is good for only about four months.

## Conclusions

The currently accepted four-month time scale for development of megaloblastic anaemia from folate deficiency, based on the early work of Herbert and others, is not consistent with the results from this study. The > 300 day liver folate storage time, predicted by the model of Lin et al., is supported by this experiment. Self-experimentation has produced a detailed record of the biochemical and haematological responses to severe experimental folate deficiency, whereas using patients or healthy volunteers as subjects would be unethical.

### Primary data

All primary data, as scanned PDF copies of pathology reports, are available from the author.

## Electronic supplementary material

Additional file 1: **Severe Experimental Folate Deficiency Part A - for Figures** 1 **to** 6 **, tables and charts.** (XLSX 435 KB)

Additional file 2: **Red-cell Survival Time, tables and charts.** (XLSX 88 KB)

Additional file 3: **Figures 1 to 6, High-resolution images.** (PDF 139 KB)

Additional file 4: **Figures 1 to 6, High-resolution slides.** (PPTX 697 KB)
