# Supplementary material for: Severe experimental folate deficiency in a human subject – a longitudinal study of biochemical and haematological responses as megaloblastic anaemia develops
Source: Springerplus. 2014 Sep 23;3:442. doi: 10.1186/2193-1801-3-442 (PMC4190280; doi:10.1186/2193-1801-3-442)
Supplement: Supplementary file 3 — Additional file 3: Figures 1 to 6, High-resolution images. (PDF 139 KB) [file 40064_2014_1258_MOESM3_ESM.pdf]

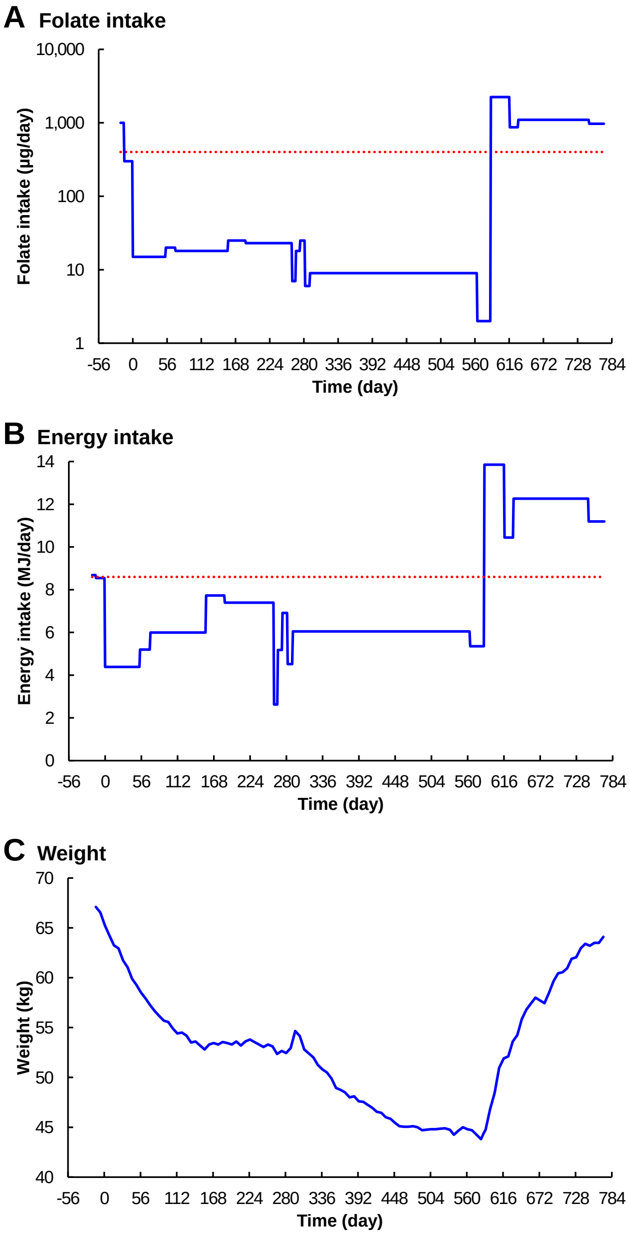
A Folate intake
Folate intake (µg/day)
10,000
1,000
100
10
1
-56 0 56 112 168 224 280 336 392 448 504 560 616 672 728 784
Time (day)
B Energy intake
Energy intake (MJ/day)
14
12
10
8
6
4
2
0
-56 0 56 112 168 224 280 336 392 448 504 560 616 672 728 784
Time (day)
C Weight
Weight (kg)
70
65
60
55
50
45
40
-56 0 56 112 168 224 280 336 392 448 504 560 616 672 728 784
Time (day)

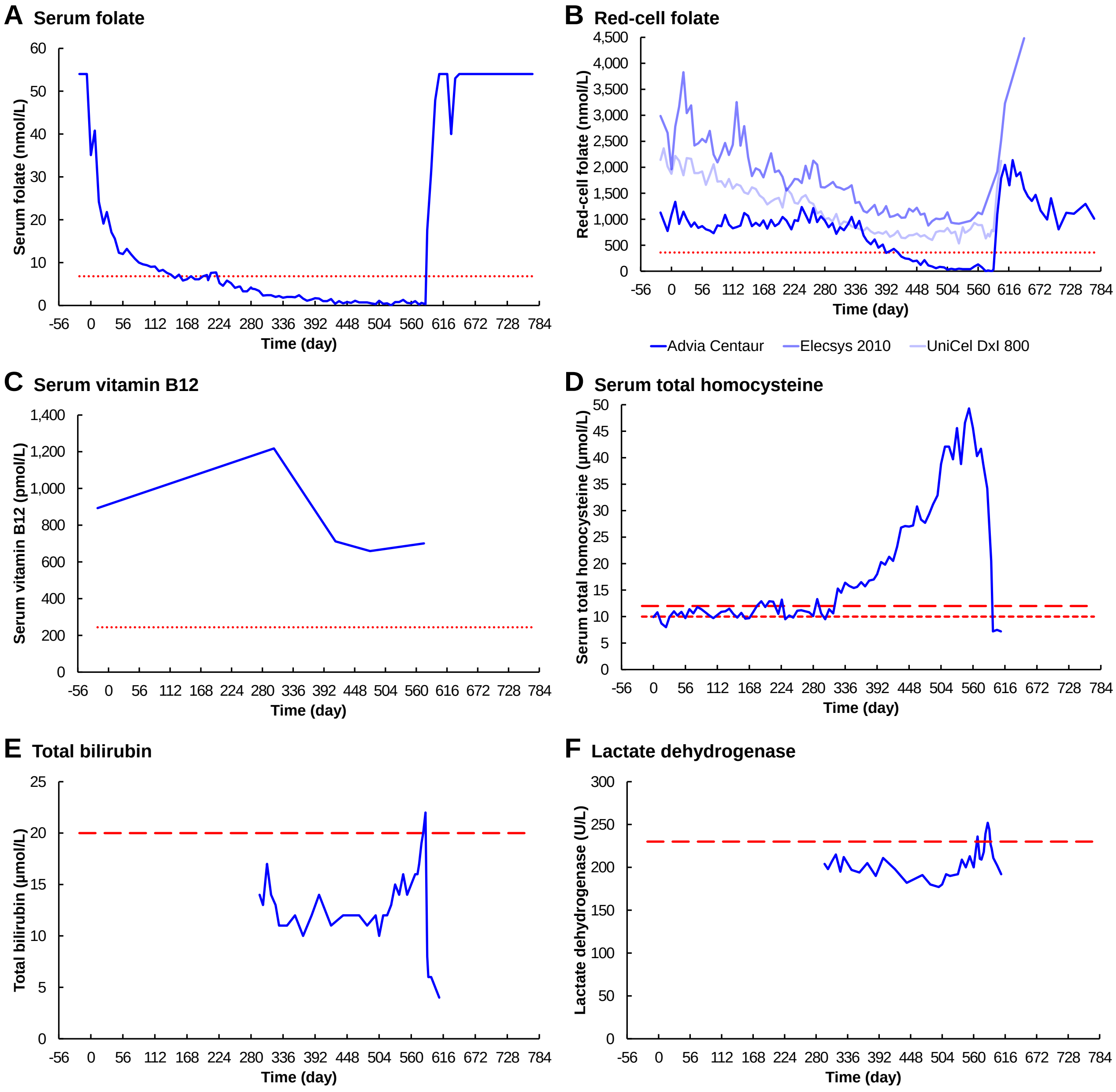
A Serum folate
Serum folate (nmol/L)
60
50
40
30
20
10
0
-56 0 56 112 168 224 280 336 392 448 504 560 616 672 728 784
Time (day)
B Red-cell folate
Red-cell folate (nmol/L)
4,500
4,000
3,500
3,000
2,500
2,000
1,500
1,000
500
0
-56 0 56 112 168 224 280 336 392 448 504 560 616 672 728 784
Time (day)
Advia Centaur
Elecsys 2010
UniCel DxI 800
C Serum vitamin B12
Serum vitamin B12 (pmol/L)
1,400
1,200
1,000
800
600
400
200
0
-56 0 56 112 168 224 280 336 392 448 504 560 616 672 728 784
Time (day)
D Serum total homocysteine
Serum total homocysteine (µmol/L)
50
45
40
35
30
25
20
15
10
5
0
-56 0 56 112 168 224 280 336 392 448 504 560 616 672 728 784
Time (day)
E Total bilirubin
Total bilirubin (µmol/L)
25
20
15
10
5
0
-56 0 56 112 168 224 280 336 392 448 504 560 616 672 728 784
Time (day)
F Lactate dehydrogenase
Lactate dehydrogenase (U/L)
300
250
200
150
100
50
0
-56 0 56 112 168 224 280 336 392 448 504 560 616 672 728 784
Time (day)

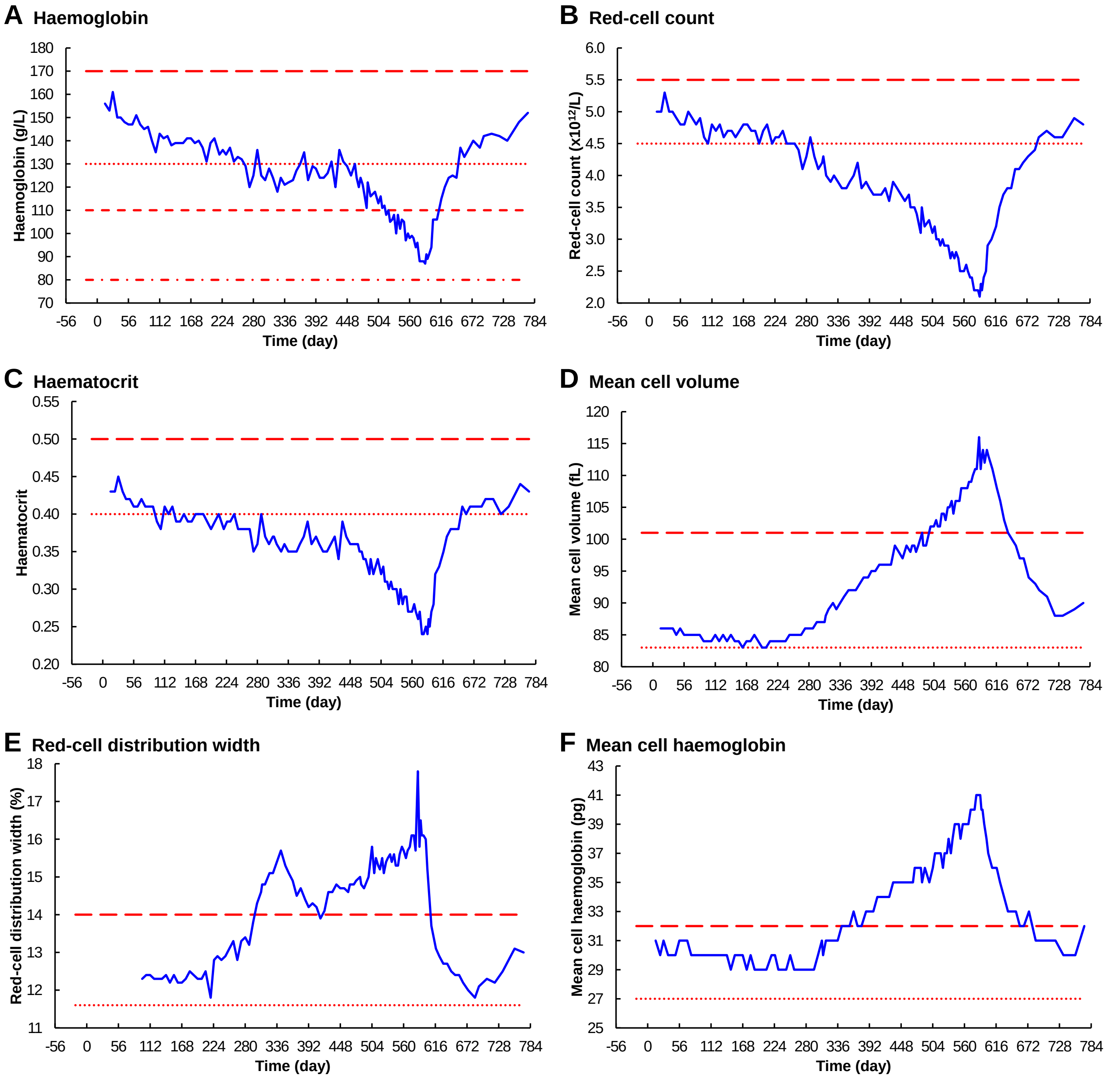
A Haemoglobin
Haemoglobin (g/L)
Time (day)
B Red-cell count
Red-cell count (x10^12/L)
Time (day)
C Haematocrit
Haematocrit
Time (day)
D Mean cell volume
Mean cell volume (fL)
Time (day)
E Red-cell distribution width
Red-cell distribution width (%)
Time (day)
F Mean cell haemoglobin
Mean cell haemoglobin (pg)
Time (day)

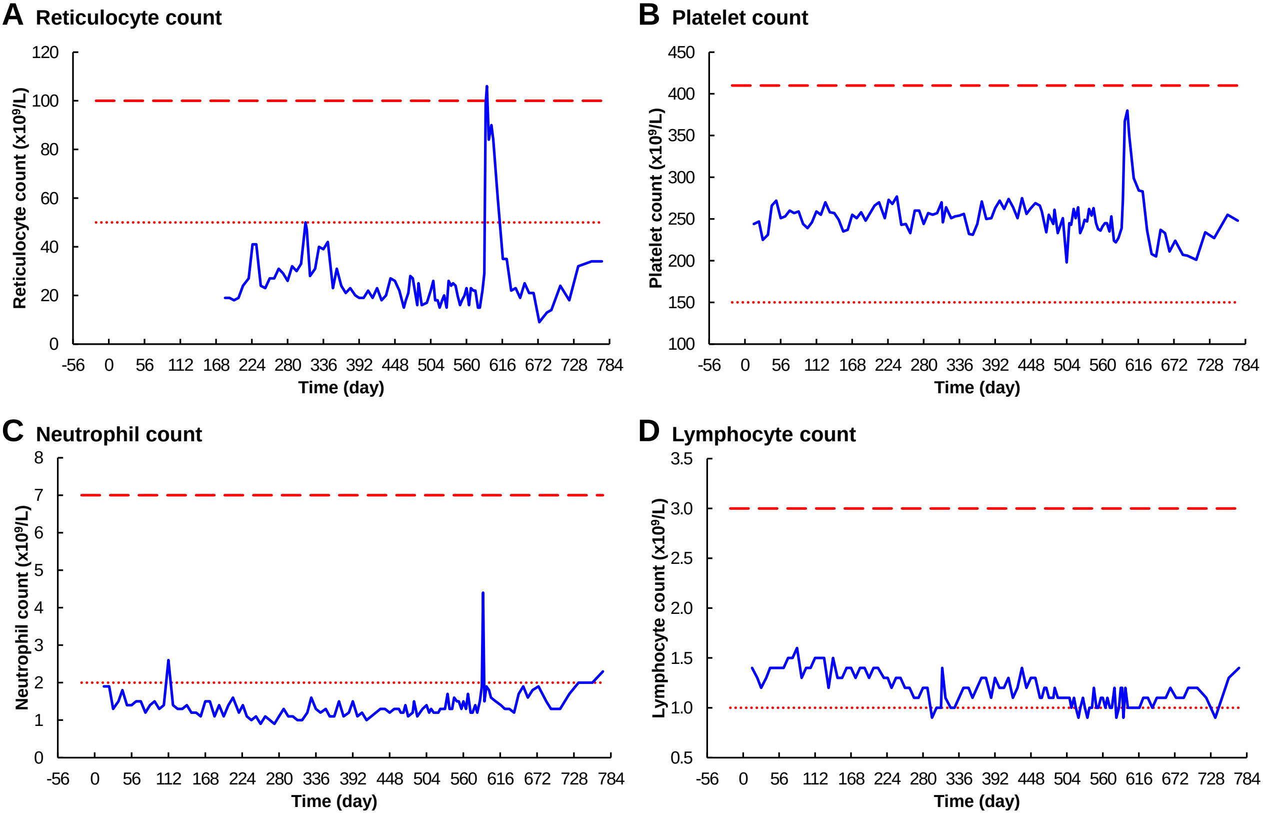
A
Reticulocyte count
Reticulocyte count (x10⁹/L)
0
20
40
60
80
100
120
Time (day)
-56
0
56
112
168
224
280
336
392
448
504
560
616
672
728
784
B
Platelet count
Platelet count (x10⁹/L)
100
150
200
250
300
350
400
450
Time (day)
C
Neutrophil count
Neutrophil count (x10⁹/L)
0
1
2
3
4
5
6
7
8
Time (day)
D
Lymphocyte count
Lymphocyte count (x10⁹/L)
0.5
1.0
1.5
2.0
2.5
3.0
3.5
Time (day)

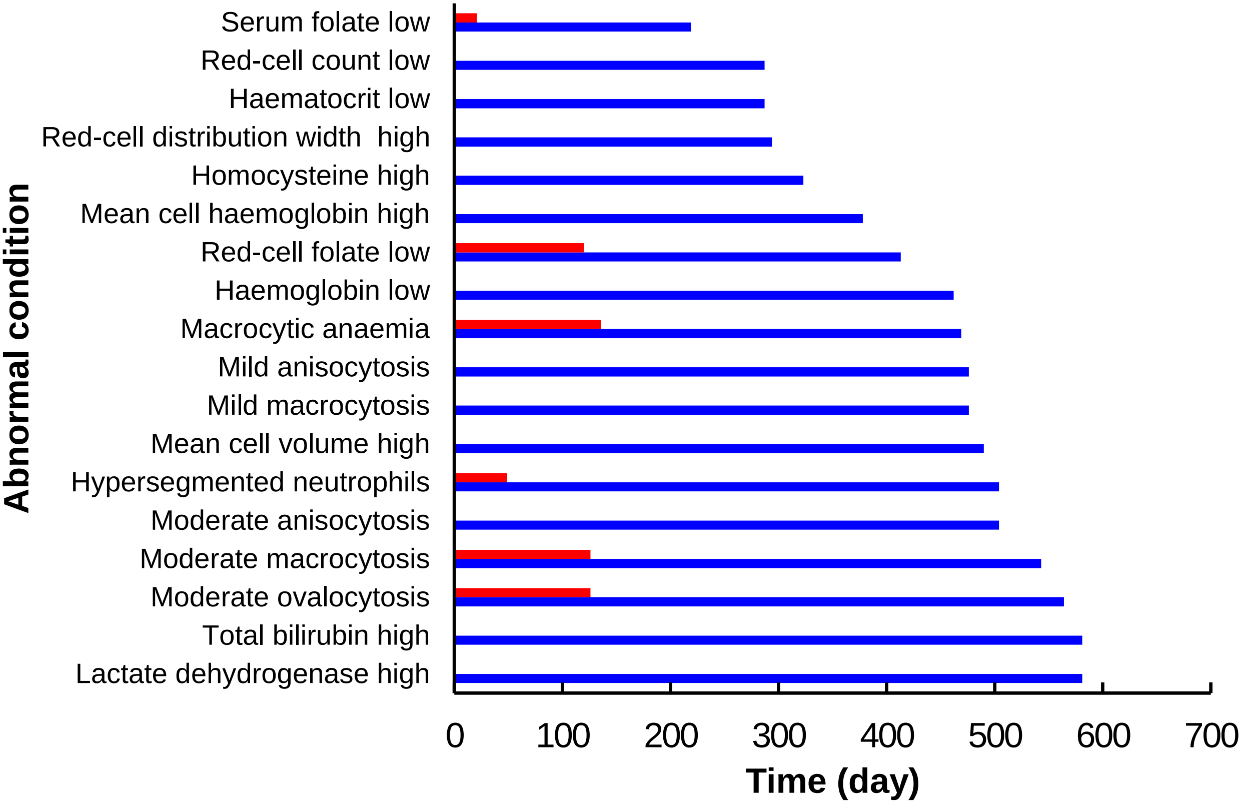
Serum folate low
Red-cell count low
Haematocrit low
Red-cell distribution width high
Homocysteine high
Mean cell haemoglobin high
Red-cell folate low
Haemoglobin low
Macrocytic anaemia
Mild anisocytosis
Mild macrocytosis
Mean cell volume high
Hypersegmented neutrophils
Moderate anisocytosis
Moderate macrocytosis
Moderate ovalocytosis
Total bilirubin high
Lactate dehydrogenase high
Abnormal condition
0
100
200
300
400
500
600
700
Time (day)

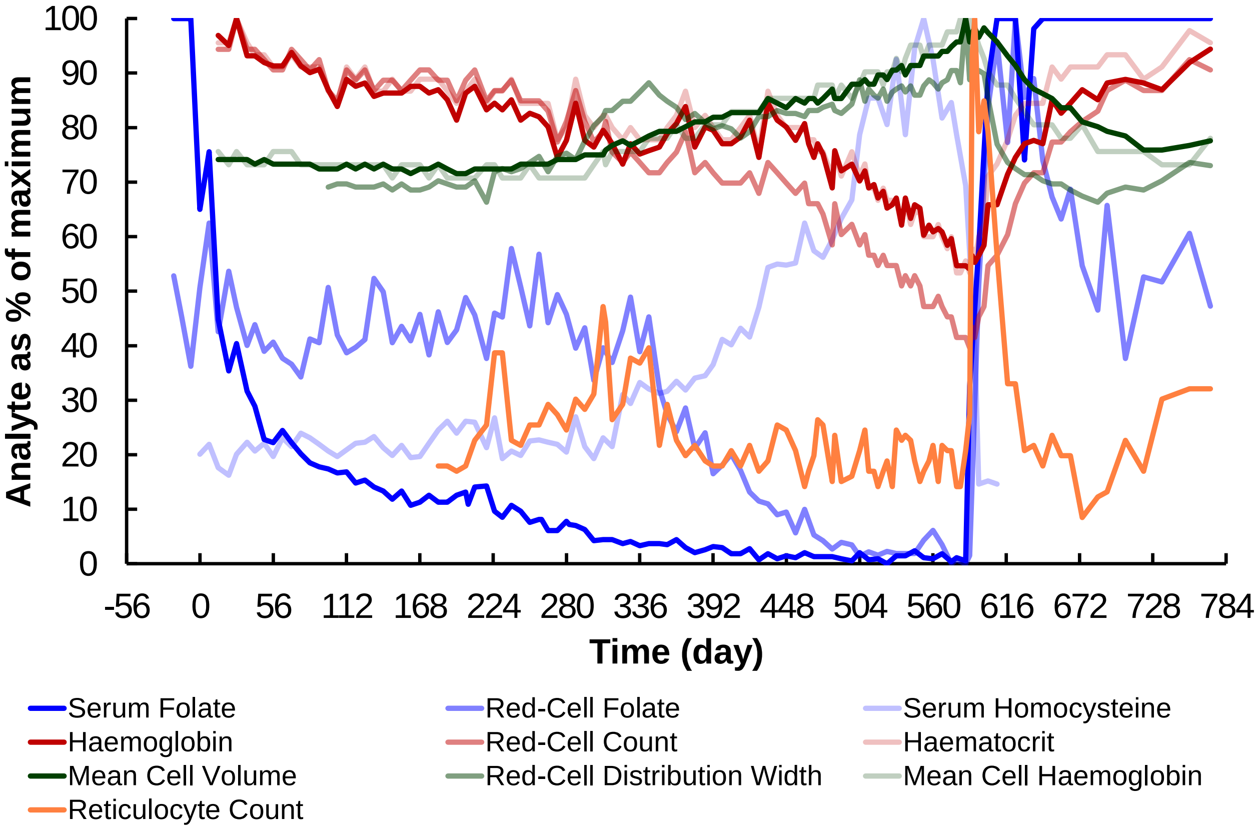

Analyte as % of maximum
100
90
80
70
60
50
40
30
20
10
0
-56
0
56
112
168
224
280
336
392
448
504
560
616
672
728
784
Time (day)
Serum Folate
Red-Cell Folate
Serum Homocysteine
Haemoglobin
Red-Cell Count
Haematocrit
Mean Cell Volume
Red-Cell Distribution Width
Mean Cell Haemoglobin
Reticulocyte Count
