# Supplementary figures and images for: Severe experimental folate deficiency in a human subject – a longitudinal study of biochemical and haematological responses as megaloblastic anaemia develops
Source: Springerplus. 2014 Sep 23;3:442. doi: 10.1186/2193-1801-3-442 (PMC4190280; doi:10.1186/2193-1801-3-442)

## Slide 1
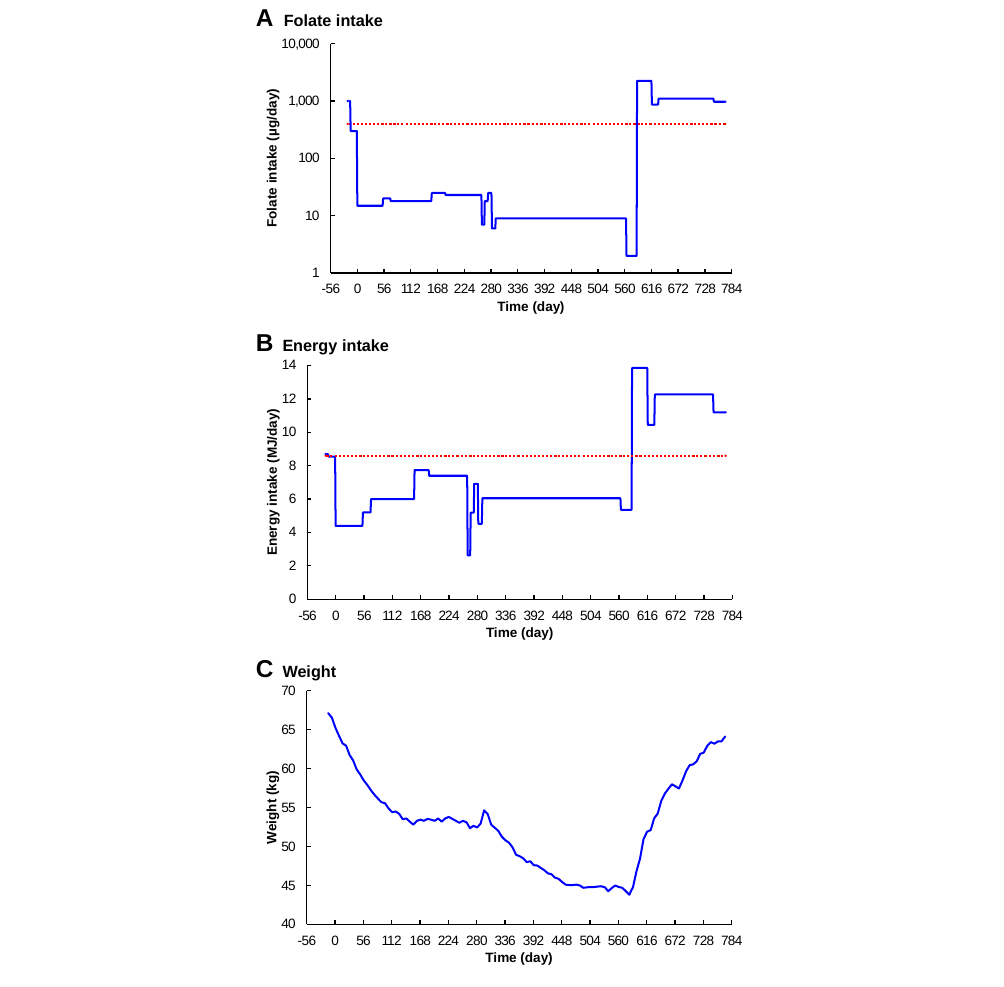

## Slide 2
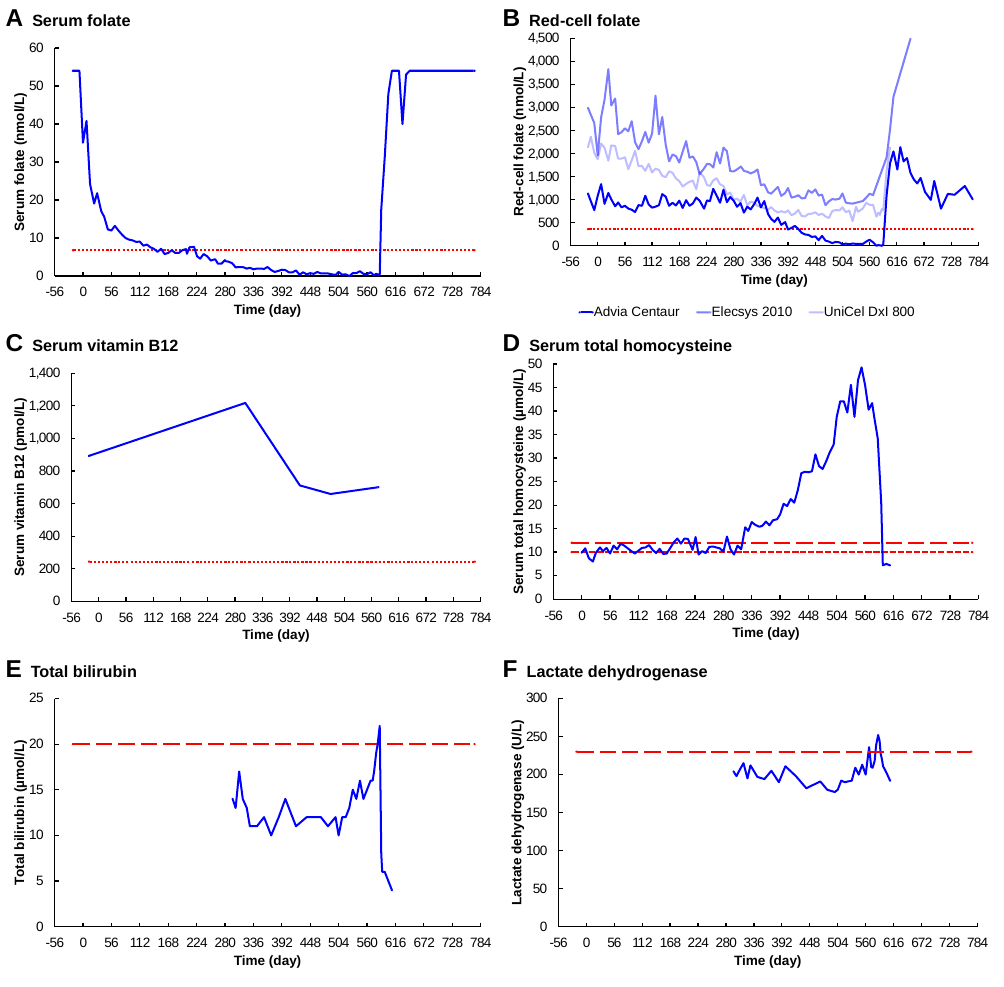

## Slide 3
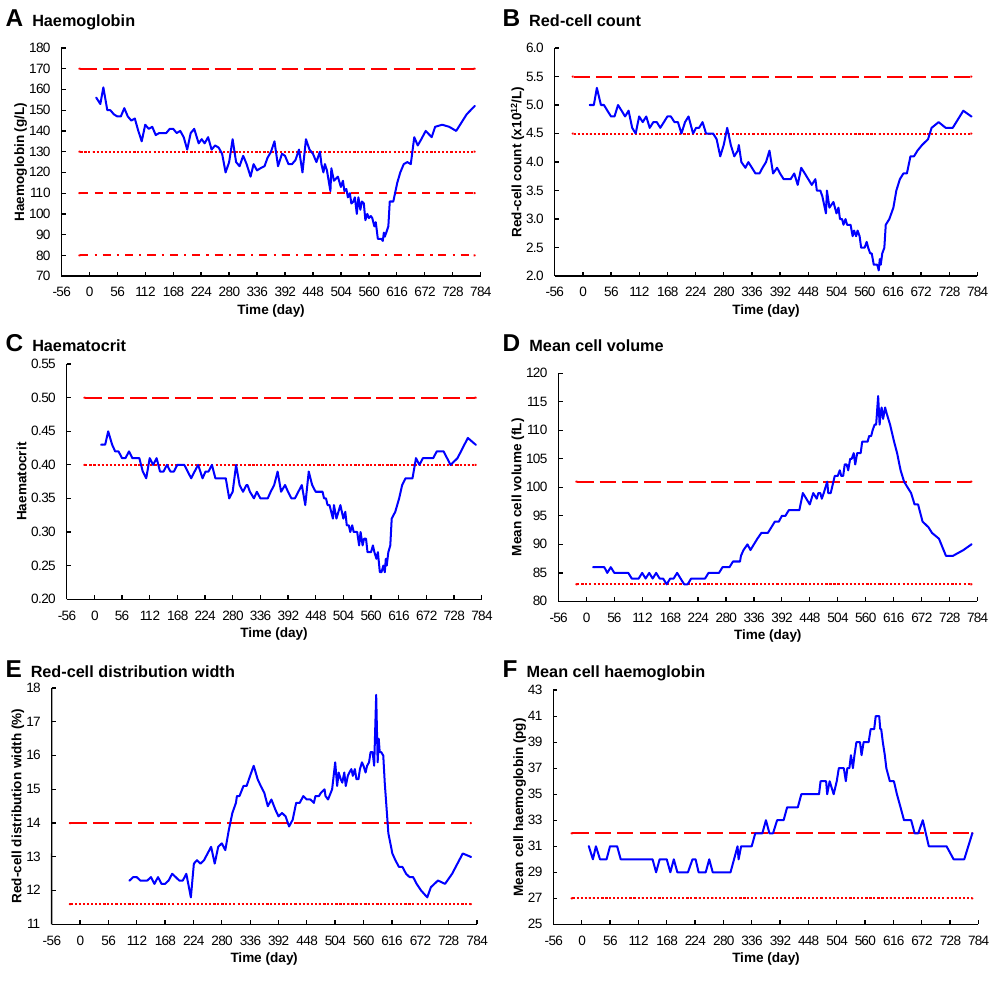

## Slide 4
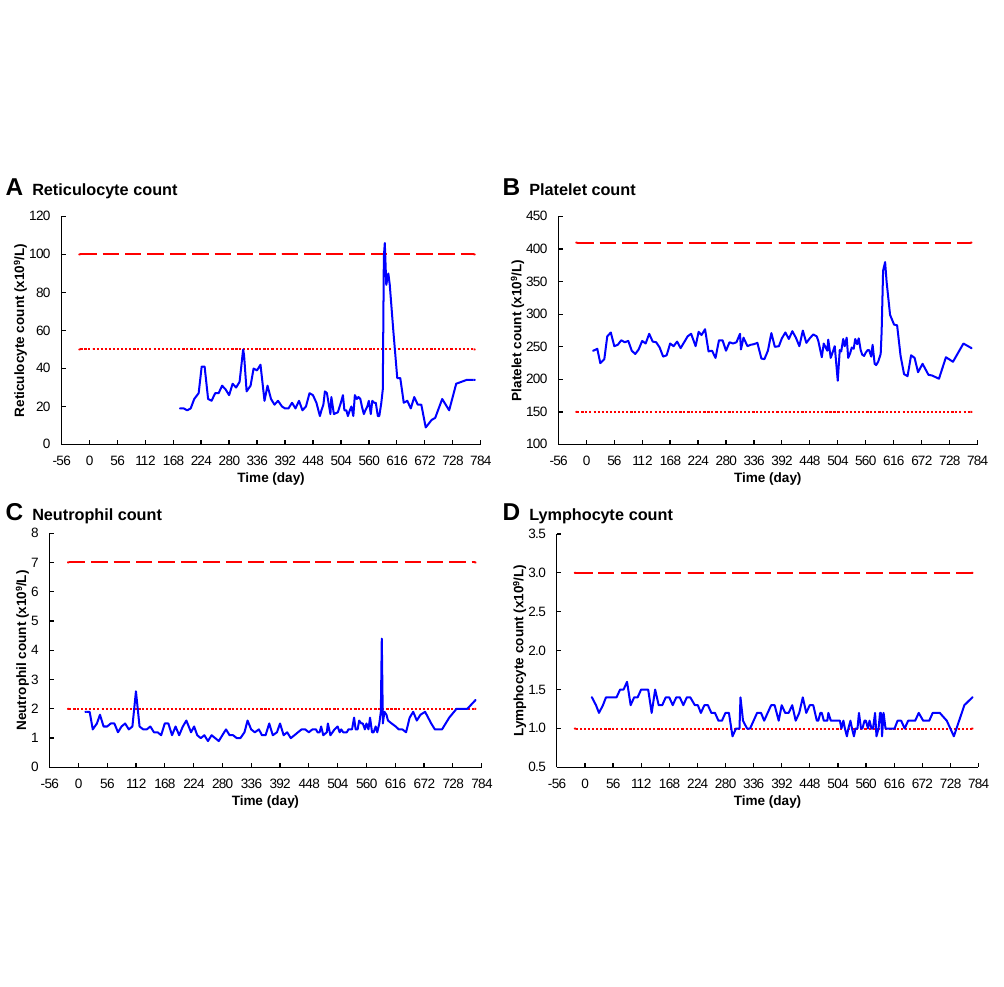

## Slide 5
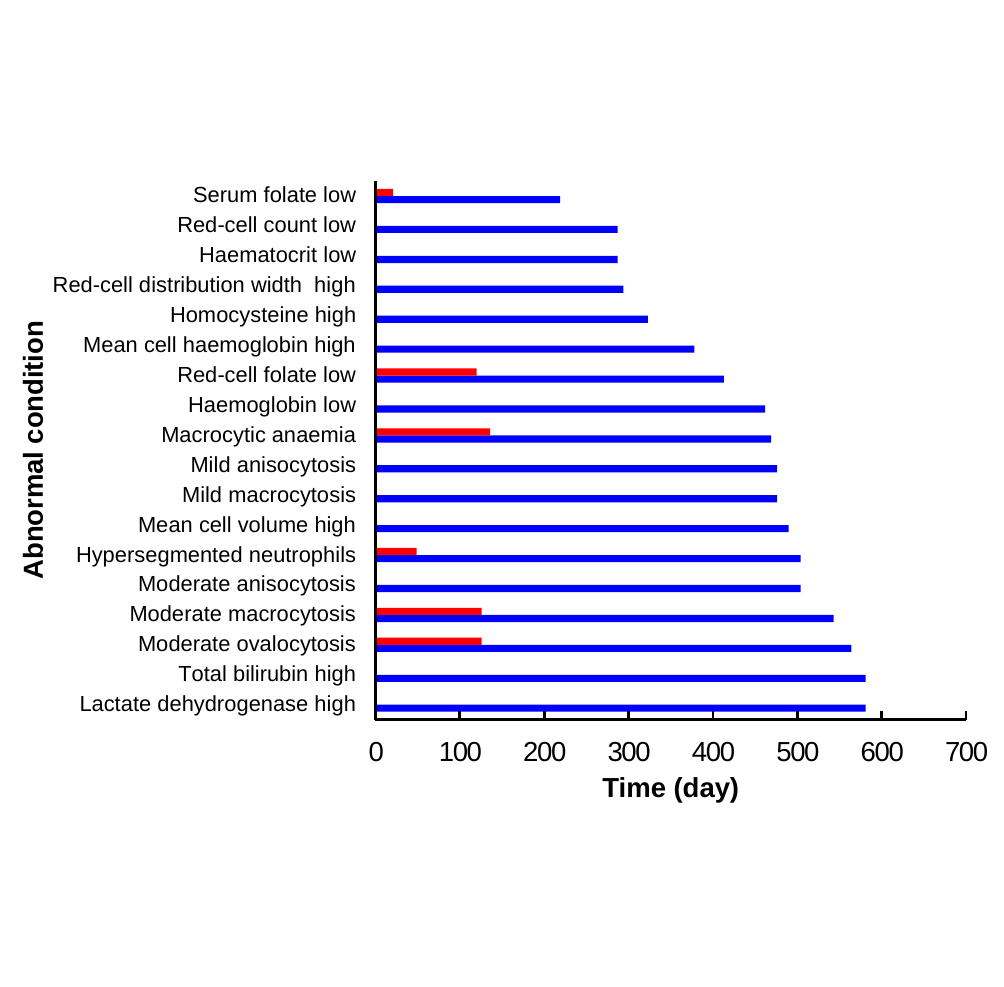

## Slide 6
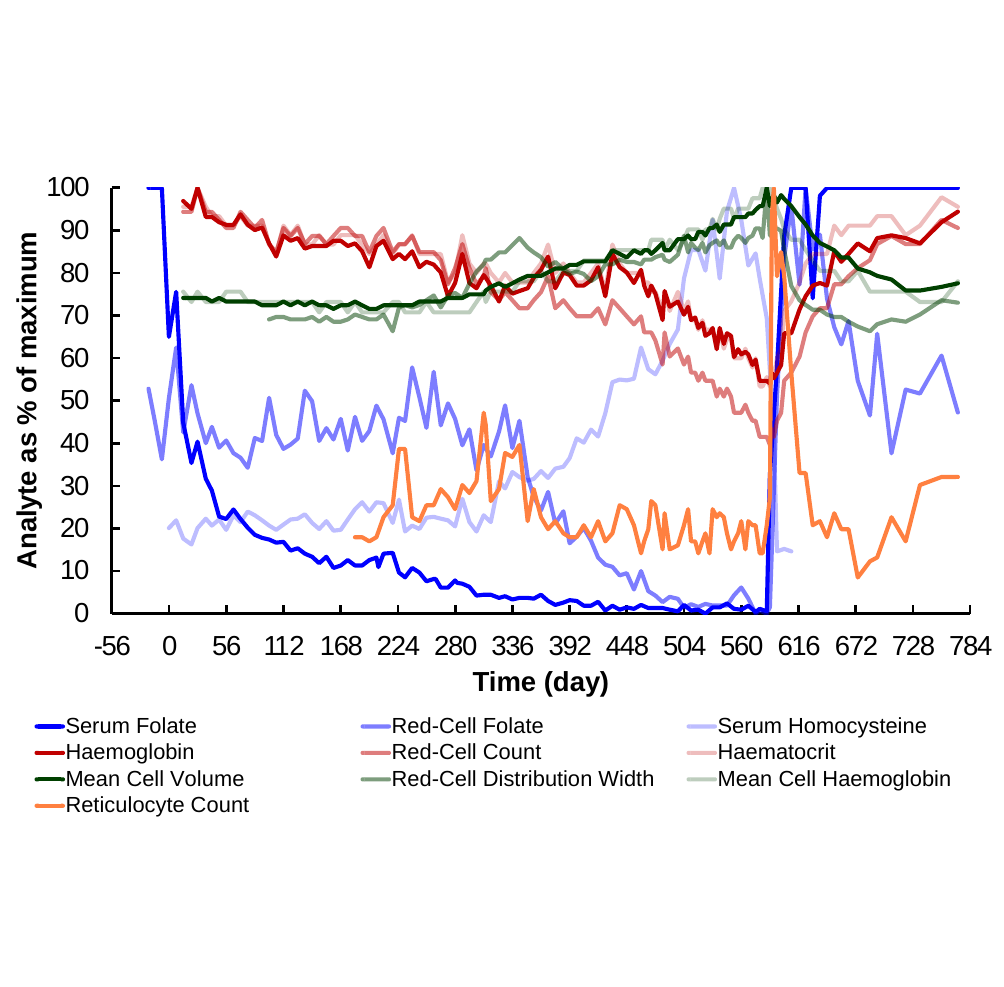

Supplement: Supplementary file 4 — Additional file 4: Figures 1 to 6, High-resolution slides. (PPTX 697 KB) [file 40064_2014_1258_MOESM4_ESM.pptx]
